# Population-based outcomes after whole brain radiotherapy and re-irradiation in patients with metastatic breast cancer in the trastuzumab era

**DOI:** 10.1186/1748-717X-6-181

**Published:** 2011-12-28

**Authors:** Irene Karam, Alan Nichol, Ryan Woods, Scott Tyldesley

**Affiliations:** 1 The Division of Radiation Oncology and Developmental Radiotherapeutics, University of British Columbia, Vancouver, BC, Canada; 2 Radiation Therapy Program, British Columbia Cancer Agency, Vancouver, BC, Canada; 3 Population Oncology, British Columbia Cancer Agency, Vancouver, BC, Canada

**Keywords:** breast cancer, brain metastasis, brain irradiation, re-irradiation, HER2 positive

## Abstract

**Purpose:**

This study examined the population-based use and outcomes of brain radiotherapy (BRT) for brain metastases (BM) from breast cancer with a focus on repeat BRT in the trastuzumab era.

**Methods and materials:**

All women with breast cancer diagnosed from 2000-2007 and treated with BRT were retrospectively identified from a provincial database.

**Results:**

A total of 441 women with BM from breast cancer were identified. The median age was 55 years and 40% (176/441) had human epidermal growth factor receptor 2 (HER2) positive disease. The median survival (MS) from the initial BRT for all 441 women was 4.5 months. The MS by Radiation Therapy Oncology Group Recursive Partitioning Analysis (RPA) class was: 1 (14.5 months), 2 (6.4 months) and 3 (1.8 months). For the 37 cases receiving repeat BRT, 27% (10/37) had stereotactic radiosurgery (SRS) and 70% (26/37) had HER2 positive disease, of which, 81% (21/26) received trastuzumab in the metastatic setting. For repeat BRT, the median survival by RPA class was: 1 (9.8 months), 2 (7.4 months) and 3 (2.0 months). For RPA class 1 and 2, the one-year overall survival (OS) was 45%.

**Conclusion:**

The proportion of cases with HER2 positive disease was increased at repeat BRT compared to initial BRT. RPA class 1 and 2 patients should be considered for repeat BRT.

## Introduction

Breast cancer is the second most common cause of BM and accounts for 5% to 15% of patients with BM [1,2]. Presenting symptoms include headaches, focal weaknesses, mental disturbances, seizures, speech difficulties, visual disturbances; any of which can impact on a patient's quality of life and length of survival [3]. Historically, median survival in patients with metastatic disease to the brain has been reported to be 3 to 4 months [4]. Younger age, presence of visceral metastases, negative estrogen receptor (ER) status and larger primary tumour size have all been associated with an increased risk of cerebral metastases [2]. In addition, the epidermal growth factor receptor 2 (HER2) has been shown to be a significant predictive and prognostic factor for the development of BM [2]. HER2 over-expression has been reported in 20%-25% of human breast cancers and is associated with a reduced overall and disease-free survival [2,5]. In an analysis of patients with invasive breast cancer referred to the British Columbia Cancer Agency (BCCA) in 2009, HER2 overexpression was identified in 16% of cases.

Studies demonstrated improved OS and progression-free survival with the use of trastuzumab in combination with chemotherapy in the setting of metastatic breast cancer in 1998 [2,6]. Improvements in disease-free survival and OS were demonstrated with trastuzumab adjuvant therapy in 2005 [7]. When the HER2 receptor is amplified in patients with breast cancer, the cancer cells tend to spread to the brain [8,9]. Improvement in the control of visceral metastasis with trastuzumab in patients with HER2 overexpression has led to longer patient survival, which increases the predilection of developing clinically apparent BM [9].

External beam whole brain radiation therapy (WBRT) is the most common local treatment for BM, followed by other treatment modalities including surgery and SRS in selected cases. Many patients with a good response to initial treatment of their BM will relapse in the brain, especially when the rest of their systemic disease is well controlled. Treatment options for recurrent cranial metastatic disease include repeat surgery, WBRT, SRS, chemotherapy and/or comfort care [8].

This is a report of a population-based study of the clinical characteristics, prognostic variables, and outcomes in patients who were treated with BRT for metastatic breast cancer in the modern era (i.e. when trastuzumab, SRS, and craniotomy for metastatic disease were available), with a special emphasis on outcomes after repeat BRT.

## Methods and materials

The BCCA provides all radiation therapy in the province of BC for a population of approximately 4.5 million. The BC Cancer Registry contains demographic data on all incident cancers, and captures date and cause of death data from death certificates. HER2 status has been tested in patients with breast cancer in BC since 1999, and trastuzumab became available for patients with metastatic breast cancer outside of a trial setting in BC in February 1999. SRS for patients with BM has been available in the province since 1998. Craniotomy for patients with BM has been practiced for decades in the province in selected cases, but became more widely practiced during the 1990's after the randomized trial by Patchell *et al *[10].

Using unique BCCA patient identifier codes, all women with breast cancer diagnosed from January 2000 to December 2007 who were treated with BRT for BM from breast cancer were identified. Among those, the patients treated with more than one course of BRT were also identified. Patients who had leptomeningeal disease (n = 7), metastatic disease to the skull (without parenchymal brain mets) (n = 55), prophylactic cranial irradiation in a clinical trial (n = 4), and male patients (n = 1) were excluded. This left 441 patients who were the subjects of the initial BRT cohort. Among those, 37 patients received a second course of BRT, three or more months after the initial course as whole brain, partial brain or SRS.

The charts of the 441 patients were reviewed and the following information was collected: age, initial stage of breast cancer, grade, hormone receptor status, HER2 status, chemotherapy use on or after the date of first BM, hormonal therapy on or after the date of first BM, trastuzumab use at any point and within 90 days of diagnosis of BM. In addition, control of primary, presence of extracranial metastases and number of brain lesions were abstracted at time of initial BRT and prior to repeat BRT. Karnofsky performance status (KPS) (≥ 70 or < 70) and the Eastern Cooperative Oncology Group (ECOG) performance status classification were identified retrospectively at initial diagnosis of BM and before repeat BRT from a description of the patient's clinical status in the chart records. A Radiation Therapy Oncology Group Recursive Partitioning Analysis (RPA) risk group [11] was determined for each patient prior to initial BRT and prior to repeat BRT. The RPA risk group used four factors: age, KPS, primary controlled/uncontrolled and extracranial metastases. Factors were inputted by the investigator for each patient and a RPA class was assigned. Patients with a KPS of < 70 were identified as class 3, patients with KPS ≥ 70, with controlled primary disease, age < 65 years and absence of extracranial metastases were identified as class 1, and all other patients as class 2 [11].

The clinical response to initial BRT and repeat BRT at 3 to 6 months from BRT was determined from the medical records. The clinical response was classified as: complete response-disappearance of neurological symptoms; partial response-alleviation of neurological symptoms; stable disease-no change in neurological symptoms; progressive disease-deterioration of neurological symptoms as described by Sadikov *et al*. [12]. The radiological response to radiation therapy at 3-6 months after initial and repeat BRT was abstracted from the radiologist's report of a post-BRT CT or MRI scan and classified as follows: complete response-disappearance of brain lesions; tumour reduction-decrease in size of brain lesions; stable disease-no change in size of brain lesions; progressive disease-increase in size and/or new brain lesions. Cases with missing information were classified as non -evaluable.

All analyses were conducted using Statistical Package for Social Sciences, version 14.0 (SPSS, Chicago, IL). OS was assessed using the Kaplan-Meier method and compared using the log-rank test. The survival time was measured from the date of starting initial BRT to the date of death or last follow-up and from the date of starting repeat BRT to the date of death or last follow-up. Patient characteristics were presented as a descriptive frequency for categorical variables. The association between each of the patient characteristics and outcomes was evaluated with univariate and multivariable analyses using a Cox regression model. This study was approved by the Research Ethics Board of the University of BC.

## Results

### Initial brain irradiation

Table 1 demonstrates the patient characteristics for all 441 women diagnosed with breast cancer and treated with initial BRT as well as the initial characteristics for those who received a second course of BRT. The median age for all 441 women was 55 years and 78% of patients were younger than 65 years (Table 1). There were 113 (26%) woman who had stage 4 disease at the time of initial diagnosis of their breast cancer. One hundred seventy six patients were HER2 positive (40%). Most women had grade 3 disease (n = 315, 71%) and had a negative ER status (n = 249, 56%). At the time of their BM diagnosis, the majority of patients (61%) had a KPS of 70 or higher. Most of the patients, 251 (57%), had multiple BM, and 344 (78%) had extracranial disease. Eighty-seven patients (20%) had a craniotomy. The RPA class of the patients was: 1 (55 patients), 2 (219 patients) and 3 (167 patients). One hundred and thirty six patients (74% of all HER2 positive patients) were treated with trastuzumab on or after the date of their first BM.

**Table 1:** Patient characteristics prior to initial brain irradiation

*Patient Characteristics*	*n = *441	*n = *37
**Age (years)**		
Median	55	48
**Grade, primary tumor**		
1	13 (3%)	1 (3%)
2	106 (24%)	13 (35%)
3	315 (71%)	22 (59%)
Unknown	7(2%)	1 (3%)
**Hormone receptor status, primary tumor**		
ER+	180 (41%)	14 (38%)
ER-	249 (56%)	22 (60%)
Unknown	12 (3%)	1 (2%)
**HER2 status, primary tumor**		
Positive	176 (40%)	26 (70%)
Negative	212 (48%)	7 (19%)
Unknown	53 (12%)	4 (11%)
**KPS at first BM**		
< 70	170 (38%)	10 (27%)
≥ 70	271 (61%)	27 (73%)
**ECOG at first BM**		
0-1	109 (25%)	21 (57%)
2	186 (42%)	13 (35%)
3-4	146 (33%)	3 (8%)
**Number of lesions**		
1-3	190 (43%)	21 (57%)
> 3	251 (57%)	16 (43%)
**Primary status**		
Controlled	305 (69%)	28 (76%)
Uncontrolled	135 (31%)	9 (24%)
**Extracranial disease**		
Brain only	97 (22%)	19 (51%)
Bone + brain	76 (17%)	6 (16%)
Visceral + brain	245 (56%)	12 (32%)
Other	23 (5%)	0 (0%)
**RPA at first BM**		
1	55 (12%)	11 (30%)
2	219 (50%)	16 (43%)
3	167 (38%)	10 (27%)
Hormonal therapy at BM*		
Yes	100 (23%)	14 (38%)
No	341 (77%)	23 (62%)
Chemotherapy at BM*		
Yes	194 (44%)	29 (78%)
No	242 (55%)	8 (22%)
Trastuzumab at BM*		
Yes	136 (31%)	21 (57%)
No	305 (69%)	16 (43%)
**Trastuzumab†**		
Yes	157 (36%)	25 (67%)
No	284 (64%)	12 (32%)
**Craniotomy**		
Yes	87 (20%)	16 (43%)
No	354 (80%)	21 (57%)

* Refers to treatments given on or after the date of first brain metastases.

†Trastuzumab use at any point including those within 90 days of brain metastases.

*Abbreviations: *ER = estrogen receptor, HER2 = human epidermal growth factor receptor 2, BM = brain metastasis, KPS = karnofsky performance status, ECOG = eastern cooperative oncology group, RPA = recursive partitioning analysis

**Figure 1:**
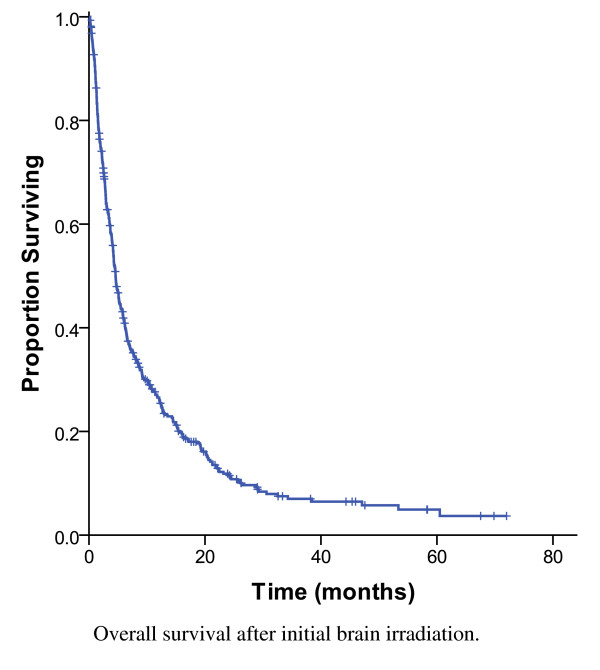
**Overall survival after initial brain irradiation, 441 patients**.

**Figure 2:**
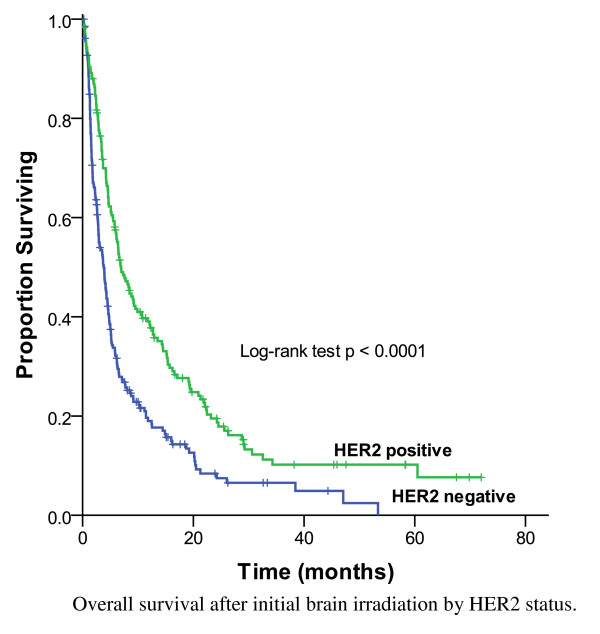
**Overall survival after initial brain irradiation by HER2 status, 441 patients**.

The two most common prescriptions for the initial WBRT were 20 Gy in 5 fractions (64%) and 30 Gy in 10 fractions (24%). WBRT was followed by a SRS boost in 4% of cases. As shown in Figure 1, the MS for all 441 subjects was 4.5 months and 26% were alive at 1 year. MS was longer (7.0 months) for the 176 subjects (40%) who had HER2 positive disease compared to those with HER2 negative disease (3.8 months, p < 0.0001) (Figure 2). The survival by RPA class was: 1 (14.5 months), 2 (6.4 months) and 3 (1.8 months) (p < 0.0001).

On univariate analysis, RPA class at diagnosis of BM (p < 0.001), SRS boost at initial BM (p < 0.001), use of craniotomy (p < 0.001), the presence of a solitary brain lesion (p < 0.001), HER2 overexpression (p < 0.001), chemotherapy use (p < 0.001), hormonal therapy use (p < 0.001) and trastuzumab therapy use (p < 0.001) at BM were all found to be favourable prognostic factors for survival (Table 2). On multivariable analysis, the only significant prognostic factors for survival were the RPA class (p < 0.001), SRS boost use (p = 0.002), craniotomy use (p < 0.001), HER2 status (p = 0.015), chemotherapy use (p < 0.001) and hormonal therapy use (p < 0.001) (Table 2).

**Table 2:** Univariate and multivariate analysis of potential prognostic factors at initial brain irradiation

	*Univariable Analysis*		*Multivariable Analysis*	
	
*Characteristics*	HR (95% CI)	*p *value	HR (95% CI)	*p *value
**RPA at diagnosis of BM**				
1	1 [Reference]		1 [Reference]	
2	1.92 (1.30-2.82)	< 0.001	1.55 (1.00-2.40)	0.05
3	5.23 (3.50-7.80)	< 0.001	4.24 (2.66-6.73)	< 0.001
**SRS boost at initial BM**				
Yes	1 [Reference]		1 [Reference]	
No	3.05 (1.71-5.44)	< 0.001	2.94 (1.51-5.73)	0.002
**Craniotomy**				
Yes	1 [Reference]		1 [Reference]	
No	2.87 (2.15-3.84)	< 0.001	2.70 (1.89-3.85)	< 0.001
**Number of lesions**				
1	1 [Reference]		1 [Reference]	
2-3	1.31 (0.91-1.89)	0.139	1.19 (0.82-1.72)	0.36
> 3	2.18 (1.66-2.88)	< 0.001	1.02 (0.78-1.34)	0.88
**HER2 status**				
Positive	1 [Reference]		1 [Reference]	
Negative and Equivocal	1.74 (1.39-2.18)	< 0.001	1.55 (1.09-2.20)	0.015
**Chemotherapy at BM**				
Yes	1 [Reference]		1 [Reference]	
No	2.26 (1.81-2.84)	< 0.001	1.81 (1.41-2.33)	< 0.001
**Hormonal therapy at BM**				
Yes	1 [Reference]		1 [Reference]	
No	1.64	< 0.001	2.22 (1.66-2.97)	< 0.001
**Trastuzumab at initial BM**				
Yes	1 [Reference]		1 [Reference]	
No	1.74 (1.37-2.20)	< 0.001	1.37 (0.94-2.01)	0.100

*Abbreviations: *RPA = recursive partitioning analysis, SRS = stereotactic radiosurgery, HER2 = human epidermal growth factor receptor 2, BM = brain metastasis

There was sufficient information available to evaluate the clinical and radiological response at 3 to 6 months after BRT in 49% (n = 218) and 75% (n = 111) of patients. A clinical improvement following initial BRT was reported in 94 patients (43%), 67 patients (31%) did not experience changes in their neurological symptoms and 57 patients (26%) deteriorated following irradiation. With respect to the radiological response, 62 patients (56%) had either complete resolution or reduction in the size of their lesions on imaging, 22 (20%) had stable disease and 27 patients (24%) had progression of their brain lesions on imaging.

### Brain re-irradiation

Table 3 demonstrates the characteristics for the 37 patients treated with repeat BRT using WBRT, partial BRT or SRS. The median age was 48 years. The median time interval from the end of the first course of BRT to repeat BRT was 29 months [4-58 months]. Most patients, 73% (27/37), had a KPS of 70 or greater prior to re-irradiation. In addition, 59% (22/37) of patients had multiple BM prior to re-irradiation, 59% (22/37) of patients had their primary breast disease controlled and 73% (27/37) of patients had extracranial disease. Among the patients who received re-irradiation, 70% (26/37) had HER2 positive disease, 57% (21/37) received trastuzumab on or after the date of the first BM and only 16% (6/37) of patients were on trastuzumab on or after progression or recurrence of the BM. All patients received a first course of WBRT for metastatic disease. The most common initial prescriptions were 20 Gy in 5 fractions (n = 21) and 30 Gy in 10 fractions (n = 14). Retreatment schemes were more variable, and included 20 Gy in 5 fractions (n = 11), 20 Gy in 10 fractions (n = 5) and 15 Gy in 5 fractions (n = 5). Ten patients (27%) received salvage SRS doses between 15 and 30 Gy in a single fraction. Seventeen patients (46%) were treated with WBRT and 12 patients (32%) received partial BRT as part of their second course of RT.

**Table 3:** Patient characteristics of 37 cases prior to re-irradiation

*Patient Characteristics prior to re-BRT*	*n = *37
**Age (years)**	
Median	48
**KPS**	
< 70	10 (27%)
≥ 70	27 (73%)
**ECOG**	
0-1	12 (32%)
2	20 (54%)
3-4	5 (13%)
**Number of lesions**	
1-3	15 (40%)
> 3	22 (59%)
**Primary status**	
Controlled	22 (59%)
Uncontrolled	15 (40%)
**Extracranial disease**	
Brain only	10 (27%)
Bone	6 (16%)
Visceral	19 (51%)
Other	2 (5%)
**RPA at re-BRT**	
1	12 (32%)
2	10 (27%)
3	15 (40%)
Chemotherapy at BM*	
Yes	19 (51%)
No	18 (49%)
Trastuzumab at BM*	
Yes	6 (16%)
No	31 (84%)

* Refers to treatments given on or after the date of brain re-irradiation.

*Abbreviations: *re-BRT = re-irradiation, RPA = recursive partitioning analysis, KPS = karnofsky performance status, ECOG = eastern cooperative oncology group performance status, BM = brain metastasis

**Figure 3:**
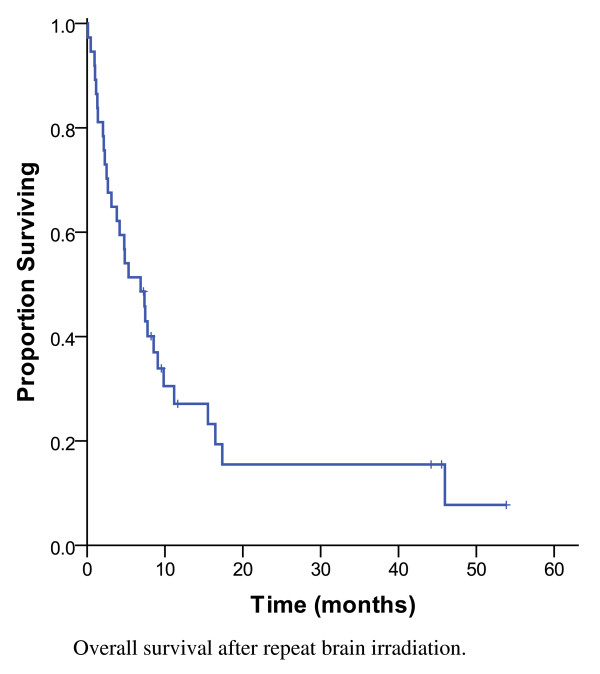
**Overall survival after repeat brain irradiation, 37 patients**.

**Figure 4:**
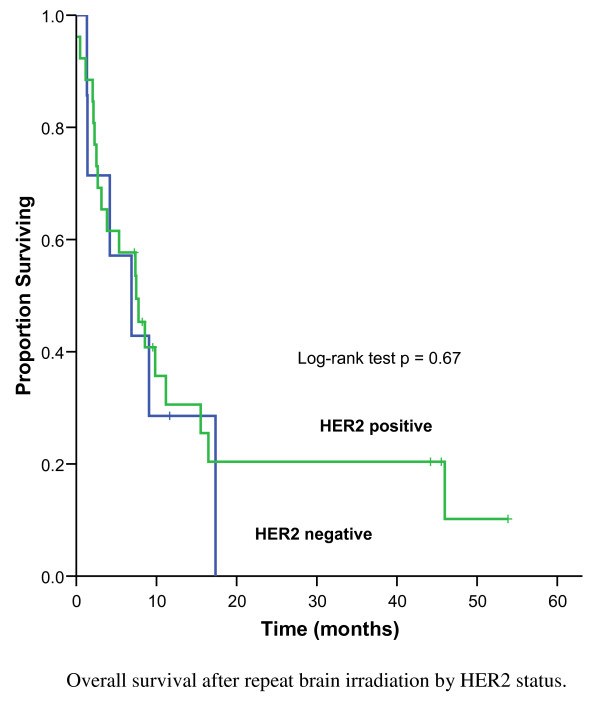
**Overall survival after repeat brain irradiation by HER2 status, 37 patients**.

**Figure 5:**
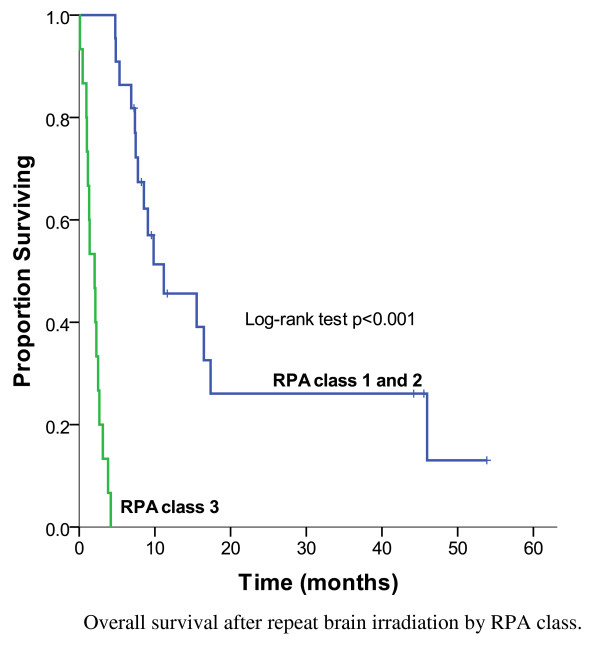
**Overall survival after repeat brain irradiation by RPA class, 37 patients**.

As shown in Figure 3, the MS after repeat BRT was 6.9 months and 14% (n = 5) were alive at 3 years. Of the re-irradiated patients with HER2 positive disease, the MS was 7.5 months compared to 6.9 months for the patients with HER2 negative disease (p = 0.67) (Figure 4). Among patients treated with salvage SRS, 30% (3/10) lived beyond 3 years. The strongest prognostic factors of patients treated with repeat BRT were the RPA class (p < 0.001), number of brain lesions at re-irradiation (p = 0.001) and tumour grade (p < 0.001) (Table 4). For patients with favourable prognostic features (RPA class 1) at the time of re-irradiation, the MS was 9.8 months, compared to 7.4 months for RPA class 2 and 2 months for RPA class 3 (Figure 5). The 1-year OS was 45% for RPA class 1 and 2, compared to 0% for RPA class 3 (p < 0.001). Following re-irradiation, 41% remained symptomatically stable, 14% had a partial clinical response and 19% had progressive disease at 3 to 6 months. Information on clinical response was not available for 26% of the repeat BRT patients.

**Table 4:** Univariate analysis of potential prognostic factors at repeat brain irradiation

*Variables*	*p *value (log-rank test)
RPA at re-BRT	< 0.001
Craniotomy at initial BM	0.18
Craniotomy at re-BRT	0.99
Number of BM at re-BRT	0.001
HER2 status	0.66
Trastuzumab on or after BM*	0.12
Chemotherapy on or after BM*	0.33
ER status	0.87
Tumour grade	< 0.001

* Refers to treatments given on or after the date of brain re-irradiation.

*Abbreviations: *re-BRT = repeat brain irradiation, RPA = recursive partitioning analysis, HER2 = human epidermal growth factor receptor 2, BM = brain metastasis, ER = estrogen receptor

## Discussion

Brain irradiation has proven useful in the treatment of BM from breast cancer [3]. Some oncologists have been reluctant to consider repeat BRT due to uncertainty about the degree of benefit and concern about the risk of severe toxicities. In recent years, many studies have reported a higher risk of central nervous system (CNS) metastases in HER2 positive patients with metastatic breast cancer [9]. The better control of extracranial disease reported in HER2 positive patients treated with trastuzumab was likely the cause of the longer survival of these CNS metastatic patients, and more cases with indications for repeat BRT. To our knowledge, this is the first population-based study describing the patterns of repeat BRT in patients with metastatic breast cancer treated in the current era. The results of population-based studies, such as this one, are more generalizable than results of randomized trials, or from single institution, tertiary referral centres.

Historically, the MS of patients with breast cancer metastatic to the brain has been poor, ranging from 3 months to 5 months [3]. In the current series of 441 patients treated with initial WBRT, the OS was 7.0 months among HER2 positive patients, with 40% of patients surviving more than 12 months. The MS for the HER2 negative patients was still only 3.8 months, which was similar to that previously reported. On a multivariable analysis, the significant prognostic factors were RPA class, SRS use, craniotomy use, HER2 status, chemotherapy use and hormonal therapy use. Several prognostic factors had been suggested in the literature, in patients treated with initial WBRT. The RTOG 79-16 study, identified KPS of 70-100, an absent/controlled primary tumour, age < 60 years, and metastatic spread limited to the brain as favourable prognostic factors for survival [13]. The prognostic value of these factors was confirmed in the current series. Patients with favourable prognostic factors (RPA class 1) had a MS of 14.5 months whereas patients with unfavourable prognostic features (RPA class 3) had a MS of 1.8 months, which was similar to the MS reported by Gaspar *et al*. (7.1 months for RPA class 1, and 2.3 months for the RPA class 3 patients) [11].

With respect to the re-irradiation cohort, the MS was 6.9 months from the start date of the second course of BRT. Historic series from other institutions have described a MS after repeat BRT ranging from 2.0 to 4.7 months for most common sites, including lung and breast cancers [12,14-18]. Most of these retrospective studies pertain to the pre-trastuzumab era, when brain re-irradiation was less frequent and systemic therapy not comparable to the current era [12,14-18]. The largest series to date, reported a MS of 4.0 months and on univariate analysis, absence of extracranial metastasis, solitary BM and a retreatment dose > 20 Gy were associated with improved survival [18].

The current study identified a subset of patients who benefited from repeat BRT in terms of survival. Patients with favourable prognostic features (RPA class 1) had a MS of 9.8 months after their second course of BRT. Another finding of these series, is the high proportion of HER2 overexpression in patients with brain involvement, as has been described by other authors [1,2,9]. Consequently, the need for local control of intracranial lesions is required, especially since for some of these patients, the probability of survival is associated with more aggressive treatment to the brain. From the BCCA's Breast Cancer Outcomes Unit database in 2009, 16% of all invasive breast cancers with known HER2 status referred to the BCCA were HER2 positive at the time of initial diagnosis, and of those with metastatic disease at the time of referral, 28% were HER2 positive. As described in the current study, 40% of those with initial BRT were HER2 positive, and of those with repeat BRT, 70% were HER2 positive. This reflects the predilection of HER2 positive disease for brain involvement particularly with better systemic control outside the brain. However, it is possible that with longer follow-up, we would have seen more HER2 negative disease recurring in the brain, and the high proportion of HER2 positive disease may reflect the aggressive early relapse in the brain in addition to a predilection for brain involvement.

This study has several limitations. First, some of the data, such as response rates and performance status were abstracted, and in some cases coded, retrospectively. This limits the strength of the response rates findings, and decreases the discrimination of our performance status categories. The beneficial effects of steroids could not be separated from the effect of irradiation due to limitations in the documentation. Data on toxicity was not abstracted due to the limitations of a retrospective design. The re-irradiation cohort, while among the largest published series of patients with breast cancer receiving repeat BRT, still had a limited number of patients who may have been highly selected from patients relapsing in the brain and this may explain the somewhat favourable survival results. Given the small re-irradiation cohort, a multivariate analysis of prognostic variables was not feasible.

Additionally, the RPA prognostic index by Gaspar *et al*. does not take into account the primary site as a parameter and the number of brain lesions. The RTOG 9508 trial showed that the number of BM was prognostic when comparing one to two or three BM [19]. A survival benefit was demonstrated for patients with a single brain lesion when treated with WBRT and SRS versus WBRT alone, whereas no survival benefit was shown for patients with multiple BM [19]. The significance of diagnosis-specific prognostic factors and indexes has been demonstrated by Sperduto *et al*. through the Graded Prognostic Assessment (GPA) [20]. The GPA uses four criteria: age, KPS, number of BM and presence of extracranial metastases to compute a score from 0.0 to 4.0, with the maximum score of 4.0 having the best prognosis [20]. In patients with breast cancer, the only significant prognostic factor found on a multivariate analysis was the KPS [20]. The current study did not use the GPA index, because the KPS was often not documented explicitly in the chart.

## Conclusion

Within the constraints of a retrospective review, this contemporary population-based study showed that the median survival after initial BRT for patients with breast cancer was 4.5 months and that, as demonstrated in previous studies, RPA class was prognostically important. Among those treated with repeat BRT, there was a higher proportion of cases with HER2 positive disease compared to HER2 negative disease. Patients with RPA class 1 and 2 should be strongly considered for brain re-irradiation.

## Competing interests

The authors declare that they have no competing interests.

## Authors' contributions

IK participated in the design of the study, performed the data collection and drafted the manuscript. AN participated in the design of the study and provided writing assistance of the manuscript. RW performed the statistical analysis. ST participated in the design of the study and data collection, performed the statistical analysis and provided writing assistance of the manuscript. All authors read and approved the final manuscript.

## References

[B1] Barnholtz-Sloan J Sloan AE Davis FG Vigneau FD Lai P Sawaya RE Incidence proportions of brain metastases in patients diagnosed (1973-2001) in the metropolitan detroit cancer surveillance system J Clin Oncol 2004 22 2865 2872 10.1200/JCO.2004.12.149 15254054

[B2] Leyland-Jones B Human epidermal growth factor receptor 2-positive breast cancer and central nervous system metastases J Clin Oncol 2009 27 5278 5286 10.1200/JCO.2008.19.8481 19770385

[B3] Tsao MN Lloyd NS Wong RK Rakovitch E Chow E Laperriere N Radiotherapeutic management of brain metastases: a systematic review and meta-analysis Cancer Treat Rev 2005 31 256 273 10.1016/j.ctrv.2005.04.007 15951117

[B4] Patchell RA Tibbs PA Regine WF Postoperative radiotherapy in the treatment of single metastases to the brain JAMA 1998 280 1485 1489 10.1001/jama.280.17.1485 9809728

[B5] Spector NL Blackwell KL Understanding the mechanisms behind trastuzumab therapy for human epidermal growth factor receptor 2-positive breast cancer J Clin Oncol 2009 27 5838 5847 10.1200/JCO.2009.22.1507 19884552

[B6] Slamon D Leyland-Jones B Shak S Addition of herceptin™ (humazined anti-HER2 antibody) to first line chemotherapy for HER2 overexpressing metastatic breast cancer (HER2+/MBC) markedly increases anticancer activity: a randomized, multinational controlled phase III trial [Abstract] Proc Am Soc Clin Oncol 1998 17 98a

[B7] Romond EH Perez EA Bryant J Trastuzumab plus adjuvant chemotherapy for operable HER2-positive breast cancer N Engl J Med 2005 353 1673 1684 10.1056/NEJMoa052122 16236738

[B8] Stemmler H Heinemann V Central nervous system metastases in HER-2-overexpressing metastatic breast cancer: a treatment challenge Oncologist 2008 13 739 750 10.1634/theoncologist.2008-0052 18614587

[B9] Lin NU Winer EP Brain metastases: the HER2 paradigm Clinical Cancer Res 2007 13 1648 1655 10.1158/1078-0432.CCR-06-2478 17363517

[B10] Patchell RA Tibbs PA Regine WF Postoperative radiotherapy in the treatment of single metastases to the brain JAMA: The Journal of the American Medical Association 1998 280 1485 1489 10.1001/jama.280.17.1485 9809728

[B11] Gaspar L Scott C Rotman M Recursive partitioning analysis (RPA) of prognostic factors in three radiation therapy oncology group (RTOG) brain metastases trials Int J Radiat Oncol Biol Phys 1997 37 745 751 10.1016/S0360-3016(96)00619-0 9128946

[B12] Sadikov E Bezjak A Yi QL Value of whole brain re-irradiation for brain metastases -- single centre experience Clin Oncol 2007 19 532 538 10.1016/j.clon.2007.06.001 17662582

[B13] Diener-West M Dobbins TW Phillips TL Nelson DF Identification of an optimal subgroup for treatment evaluation of patients with brain metastases using RTOG study 7916 Int J Radiat Oncol Biol Phys 1989 16 669 673 10.1016/0360-3016(89)90483-5 2646260

[B14] Abdel-Wahab MM Wolfson AH Raub W The role of hyperfractionated re-irradiation in metastatic brain disease: a single institutional trial Am J Clin Oncol 1997 20 158 160 10.1097/00000421-199704000-00011 9124191

[B15] Cooper JS Steinfeld AD Lerch IA Cerebral metastases: value of reirradiation in selected patients Radiol 1990 174 883 885 10.1148/radiology.174.3.2305074 2305074

[B16] Hazuka MB Kinzie JJ Brain metastases: results and effects of re-irradiation Int J Radiat Oncol Biol Phys 1988 15 433 437 10.1016/S0360-3016(98)90026-8 2841266

[B17] Kurup P Reddy S Hendrickson FR Results of re-irradiation for cerebral metastases Cancer 1980 46 2587 2589 10.1002/1097-0142(19801215)46:12<2587::AID-CNCR2820461209>3.0.CO;2-4 7448697

[B18] Wong WW Schild SE Sawyer TE Shaw EG Analysis of outcome in patients reirradiated for brain metastases Int J Radiat Oncol Biol Phys 1996 34 585 590 10.1016/0360-3016(95)02156-6 8621282

[B19] Andrews DW Scott CB Sperduto PW Whole brain radiation therapy with or without stereotactic radiosurgery boost for patients with one to three brain metastases: phase III results of the RTOG 9508 randomised trial Lancet 2004 363 1665 1672 10.1016/S0140-6736(04)16250-8 15158627

[B20] Sperduto PW Chao ST Sneed PK Diagnosis-specific prognostic factors, indexes, and treatment outcomes for patients with newly diagnosed brain metastases: a multi-institutional analysis of 4,259 patients Int J Radiat Oncol Biol Phys 2010 77 655 661 10.1016/j.ijrobp.2009.08.025 19942357

